# Response to letter to the editor entitled: “Correlation analysis of the triglyceride glucose index and heart failure with preserved ejection fraction in essential hypertensive patients”

**DOI:** 10.1002/clc.23943

**Published:** 2022-11-20

**Authors:** Li Ping Liao, Yang Yang

**Affiliations:** ^1^ Department of Cardiology Jiading Branch of Shanghai General Hospital, Shanghai Jiao Tong University School of Medicine Shanghai China; ^2^ Department of Cardiology The First Affiliated Hospital of Anhui Medical University Hefei Anhui China

**Keywords:** heart failure with preserved ejection fraction, hypertension, TyG index

1

To the Editor,

We welcome and appreciate the comments raised by Mei et al. related to our recent publication “Correlation analysis of the triglyceride glucose index and heart failure with preserved ejection fraction in essential hypertensive patients.” In the article, we analyzed the correlation between TyG index and occurrence of heart failure with preserved ejection fraction (HFpEF) in hypertensive patients. Our findings demonstrated TyG index is higher in HFpEF patients than in nonHFpEF patients and related to cardiac diastolic function, which strongly correlates with occurrence of HFpEF in hypertensive patients.

In response to the comments raised in this letter, we present a few clarifications here.

As described in the exclusion criteria of our article, all enrolled patients did not use drugs that affect blood glucose before admission, which means that all enrolled patients were not diagnosed with diabetes before admission, we could not get the data of diabetes history during the collection of previous medical history. In the grouping of clinical data of the enrolled patients, we found that there was no statistical significant difference in the number of patients diagnosed with diabetes between the two groups after this admission, so we did not mention it further in the article. If the author wants to explore the role of TyG index on cardiovascular disease in nondiabetic population, we think that the results of only including nondiabetic patients as research objects are more convincing.

In terms of the gender of the two groups of patients, it has been shown in the article that there is no significant statistical difference between the two groups. After reviewing the relevant literature, we found that whether there is a gender difference between the TyG index and the risk of major adverse cardiovascular events is inconclusive. Only in terms of the interaction between the TyG index and gender in predicting myocardial infarction, the prediction efficiency of the TyG index for women is higher. After consulting relevant data, we believe that this gender difference can be explained by the increased insulin resistance of menopausal women with decreased estrogen levels.^1^ However, as mentioned by the author, the average age of our study population is relatively higher, and most of the female patients are menopausal, this might weaken the gender differences caused by menopausal women. As the author mentioned, Wu et al.^2^ also suggested in their research on “Triglyceride and Glucose Index and Sex Differences in Relation to Major Adverse Cardiovascular Events in Hypertensive Patients Without Diabetes”: After restricted cubic splines were used to flexibly model and visualize the relationship between the TyG index and MACEs.” “There was no interaction between the sex and TyG index (*p* for interaction = .73).” In conclusion, they found that TyG index was associated with MACEs in hypertensive patients, and there was no gender difference between the TyG index and MACEs. Therefore, we do not think that further discussion is necessary.

We are afraid that the author does not really understand the research point of our article; this study is a retrospective study only for patients with essential hypertension, the results of our study are only applicable to people with essential hypertension, and cannot be expanded to general population. It is precisely because a large number of literature have reported that TyG index is related to cardiovascular and cerebrovascular diseases in the past. On this basis, we have an idea to further study whether TyG index is related to heart failure with preserved ejection fraction in essential hypertensive patients. In the discussion, we also mentioned that the mechanism of TyG index in is not clear. More studies on mechanism are still needed in the future.

The area under the curve (AUC) predicted by the TyG index for the incidence of heart failure is different between Zeng et al.^3^ and our study. We think that this may be related to the different research objects of the two studies. Zeng et al.^3^ based their research on African Americans and Whites aged 18–30. The study group is young and has a relatively low probability of having basic diseases. Our study group is the Chinese elderly with essential hypertension, and also the probability of having basic diseases is higher than in the young group. Cardiac structure changes occur in patients with essential hypertension, most of them have experienced a long course of disease progression. Therefore, our study population is older. We believe that it is reasonable to have differences in AUC values based on different races and different age groups. However, the research results are consistent: Both suggest that TyG index is related to the occurrence of heart failure.

## CONFLICT OF INTEREST

None.

## Data Availability

None declared.

## References

[1] Lovejoy JC , Champagne CM , de Jonge L , Xie H , Smith SR . Increased visceral fat and decreased energy expenditure during the menopausal transition. Int J Obes. 2008;32(6):949‐958. 10.1038/ijo.2008.25 PMC2748330 18332882

[2] Wu Z , Liu L , Wang W , et al. Triglyceride‐glucose index in the prediction of adverse cardiovascular events in patients with premature coronary artery disease: a retrospective cohort study. Cardiovascular Diabetology. 2022;21(1):142. 10.1186/s12933-022-01576-8 35906587 PMC9338459

[3] Zeng X , Han D , Zhou H , et al. Triglyceride‐glucose index and homeostasis model assessment‐insulin resistance in young adulthood and risk of incident congestive heart failure in midlife: the coronary artery risk development in young adults study. Front Cardiovasc Med. 2022;9:944258. 10.3389/fcvm.2022.944258 35845059 PMC9279654

